# Integration of Artificial Intelligence in Endometrial Cancer Management

**DOI:** 10.7759/cureus.114356

**Published:** 2026-08-11

**Authors:** Fatima Safini, Sanae Abbaoui, Slimane Semghouli, Bouchra Amaoui

**Affiliations:** 1 Biotechnology and Medicine (BioMed) Laboratory, Faculty of Medicine and Pharmacy, Ibn Zohr University, Agadir, MAR; 2 Radiation Oncology, Mohammed VI University Hospital Center, Agadir, MAR; 3 Medical Physics, Higher Institute of Nursing Professions and Health Techniques, Agadir, MAR

**Keywords:** artificial intelligence, deep learning, digital pathology, endometrial cancer, machine learning, precision oncology, prognostic prediction, radiomics, risk stratification

## Abstract

Endometrial cancer is one of the most frequent gynecologic malignancies in developed countries, with increasing incidence worldwide. The complexity of molecular and clinicopathologic data has highlighted the need for advanced analytical tools to optimize risk assessment and therapeutic decision-making. Artificial intelligence (AI) has emerged as a disruptive technology in oncology, enabling high-dimensional data integration and predictive modeling. Its application in endometrial cancer holds significant potential to improve diagnosis, prognostic stratification, and individualized treatment strategies. This narrative review synthesizes evidence from original research articles, systematic reviews, meta-analyses, and emerging translational studies focusing on AI applications in endometrial cancer. It evaluates AI-driven approaches in screening and early detection, machine learning-based diagnostic and risk stratification models, radiomics and imaging analytics, deep learning applications in histopathology and molecular classification, and predictive algorithms for treatment planning and prognostic assessment. A marked increase in scientific publications over the past decades underscores the expanding role of AI in endometrial cancer research. AI-based systems leveraging multimodal data including clinical variables, imaging radiomics, digital histopathology, and molecular profiling demonstrate improved performance in diagnostic classification, risk prediction, and treatment optimization. Enhanced accuracy in tumor grading, molecular subtype prediction, and survival modeling compared with conventional statistical approaches was reported. AI represents a real opportunity to enhance the comprehensive management of endometrial cancer. Nevertheless, its clinical adoption depends on rigorous external validation, harmonization of datasets, transparency of algorithms, and integration into multidisciplinary decision-making pathways that preserve clinical judgment and ensure equitable access to innovation.

## Introduction and background

Endometrial cancer (EC) is the sixth most common malignancy among women worldwide and the second most frequent gynecologic cancer after cervical cancer. In 2022, an estimated 420,242 new cases were diagnosed globally, accounting for approximately 4.3% of all cancers in women, with 97,704 associated deaths [1]. Its rising incidence, particularly in high-income countries, has been partly attributed to increasing obesity rates and population aging, highlighting the need for improved risk stratification and personalized management strategies [2]. Artificial intelligence (AI), a rapidly evolving data-driven discipline encompassing machine learning, deep learning, and advanced computational modeling, has emerged as a promising tool to address complex challenges in modern healthcare. In oncology, AI applications have demonstrated significant potential in enhancing diagnostic accuracy, optimizing treatment planning, and improving prognostic prediction, particularly in cancers such as lung and breast cancer. Despite these advances, the role of AI in the comprehensive management of EC remains comparatively underexplored, revealing an important gap in the current literature [3-5]. Through this comprehensive review, we aim to synthesize emerging evidence on the role of AI in the management of EC. We analyze AI-driven applications in screening, diagnostic workflows, molecular and histopathological classification, risk stratification, and treatment optimization. This review highlights AI’s potential to advance precision oncology in EC, its applicability, and its limitations.

## Review

Methodology

This article was conducted as a narrative review aiming to provide an updated overview of the current and emerging applications of AI in EC management. A literature search was performed using the PubMed database to identify relevant publications available up to April 2025. The search strategy included combinations of the following keywords: "artificial intelligence", "machine learning", "deep learning", "endometrial cancer", "screening", "diagnosis", "radiomics", "histopathology", "molecular classification", "treatment", and "prognosis". The retrieved articles were screened for their relevance to the objectives of this review. Priority was given to original studies, systematic reviews, and meta-analyses reporting clinically meaningful applications of AI in EC. Publications were selected based on their scientific relevance, methodological quality, and contribution to one or more stages of the clinical management pathway. The selected literature was narratively synthesized and organized into five major domains: screening, diagnosis, staging, treatment, and prognosis. Because this review was designed as a narrative synthesis rather than a systematic review, no formal risk-of-bias assessment or quantitative meta-analysis was performed.

Application of AI in screening

Currently, no validated population-based screening strategy exists for EC. Although several approaches including transvaginal ultrasound, endometrial cytology, and genetic testing have been investigated, their diagnostic performance remains insufficient for routine screening due to limited sensitivity and lack of cost-effectiveness. Recent studies have highlighted the potential of AI-based models for the development of non-invasive screening strategies in EC. Troisi et al. developed a rapid and cost-effective diagnostic approach based on a serum metabolomic signature capable of identifying EC prior to symptom onset [3]. To further validate this strategy, the authors applied a machine learning algorithm to a cohort of 1,550 postmenopausal women. The model demonstrated excellent diagnostic performance, with sensitivity and specificity exceeding 99%. Despite these promising findings, external validation in larger and more diverse populations is required before clinical implementation can be considered [4]. Other predictive models have been assessed to stratify women into high-risk groups, which would allow for the implementation of preventive strategies in this population. In a study published in 2020, researchers developed seven machine learning algorithms (logistic regression (LR), neural network (NN), support vector machine (SVM), decision tree (DT), random forest (RF), linear discriminant analysis (LDA), and naïve Bayes (NB)) to predict the five-year risk of developing EC. The proposed models were constructed solely from clinical and lifestyle-related data, without incorporating multimodal inputs such as genomic, imaging, or histopathological features. RF and NN architectures achieved superior predictive accuracy compared with other machine learning techniques. These findings suggest that AI-driven risk models could enhance individualized risk assessment and guide preventive and screening strategies in high-risk women before the development of EC. Nevertheless, validation across diverse populations and integration into clinical workflows remain essential before routine implementation [5]. Several AI-driven models have sought to identify high-risk women who may require intensified surveillance. As a scalable and non-invasive approach, AI has the potential to enhance individualized risk assessment and support preventive strategies in EC. Early identification of high-risk subgroups could enable the development of standardized monitoring pathways incorporating clinical evaluation and imaging modalities, currently lacking in routine screening frameworks. In addition, such stratification may allow for tailored preventive interventions targeting modifiable lifestyle factors, including obesity reduction, increased physical activity, and dietary optimization [6]. A recent systematic review and meta-analysis including 13 studies evaluated the diagnostic performance of AI in EC screening. The pooled analysis reported a sensitivity of 86% (95% CI: 79%-90%) and a specificity of 92% (95% CI: 87%-95%), suggesting promising diagnostic accuracy across different AI models and study settings. However, the overall quality of evidence was rated as low, highlighting the need for large-scale population-based validation studies before routine clinical implementation [7]. Future screening paradigms for EC may integrate AI-based digital tools capable of analyzing self-reported and clinical health information to generate personalized risk predictions. By enabling proactive risk stratification prior to symptom onset, these systems could guide targeted surveillance and preventive interventions. With robust validation and appropriate regulatory oversight, such innovations may contribute to a paradigm shift in EC prevention and early detection.

Applicability of AI in diagnosis

EC predominantly affects postmenopausal women, with fewer than 5% of cases diagnosed in women under the age of 40. Postmenopausal bleeding remains the most common and clinically significant presenting symptom. However, during the perimenopausal period, differentiating pathological bleeding from physiological menstrual irregularities can be particularly challenging [8]. In such situations, the diagnostic workup typically includes gynecological examination, hysteroscopy, and imaging modalities such as transvaginal ultrasound and pelvic magnetic resonance imaging (MRI). Definitive diagnosis relies on histopathological evaluation of endometrial tissue obtained through dilation and curettage (D&C) or Pipelle biopsy [9]. Given the complexity and variability of these diagnostic pathways, an important question emerges: can AI enhance diagnostic accuracy by optimizing clinical assessment, imaging interpretation, and histopathological analysis in EC [10]? A study published in 2023 by Erdemoglu et al. analyzed data from 564 women presenting with abnormal vaginal bleeding, aiming to develop a simple and clinically applicable AI model for the diagnosis of EC. Several clinico-epidemiological variables were initially evaluated; however, feature selection using the Boruta algorithm identified only three key predictors for inclusion in the final model. Body mass index (BMI), age, and endometrial thickness were retained as the most relevant variables, given their strong association with precancerous lesions and EC. These findings suggest that AI-based models relying on readily available clinical parameters may assist in identifying women at increased risk of endometrial intraepithelial neoplasia or malignancy in the context of abnormal uterine bleeding [11]. AI could predict EC risk in postmenopausal women with abnormal bleeding when endometrial thickness is ≥5 mm, as evaluated by a simple transvaginal ultrasound performed in primary healthcare centers [12]. Five studies addressed AI applications in ultrasound-based diagnosis of EC, reporting encouraging results in diagnostic classification and histopathological correlation. However, methodological limitations and lack of external validation limit their immediate clinical applicability. In a literature review exploring the role of AI applied to ultrasound imaging in gynecologic oncology, 50 studies were included. Among them, five specifically investigated AI applications in the ultrasound-based diagnosis of EC and reported encouraging results in diagnostic classification and histopathological correlation. However, methodological limitations and the lack of external validation restrict their immediate clinical applicability [13]. Given the role of hysteroscopy as a second-line diagnostic tool in women with abnormal uterine bleeding, the integration of deep learning models into hysteroscopic image analysis represents a logical extension of AI applications in EC detection. Zhang et al. assessed the impact of AI in classifying endometrial lesions visualized through hysteroscopy in women with abnormal bleeding. They collected 1,851 images from 454 patients with endometrial lesions observed via hysteroscopy. The images were preprocessed and fed into a fine-tuned VGGNet-16 model. The authors then compared AI-based diagnoses with those established by gynecologists. They concluded that the model slightly outperformed gynecologists in classifying endometrial lesions. This study validates the feasibility and applicability of deep learning in assisting clinical diagnosis of endometrial lesions based solely on hysteroscopic images [14]. Imaging plays a central role in both the diagnosis and pre-therapeutic staging of EC. Transvaginal and/or pelvic ultrasound is generally used as the first-line examination, while pelvic MRI remains the gold standard for locoregional staging. Radiology represents one of the medical specialties in which AI has demonstrated substantial progress. In this context, AI aims to reduce radiologists’ workload, minimize diagnostic errors and interobserver variability, and enhance overall diagnostic accuracy. By automating image analysis and extracting complex patterns, AI systems may facilitate faster and more efficient diagnostic workflows. Radiomics, a rapidly evolving field within imaging research, involves the extraction of high-dimensional quantitative features from radiological images and their integration with clinical and biological data to support diagnosis and risk stratification. A recent literature review evaluating the role of radiomics in the preoperative assessment of EC included 17 studies investigating AI-based imaging approaches, including machine learning and deep learning models. The authors concluded that the current level of evidence remains insufficient to validate the routine clinical use of radiomics in EC [15]. Similarly, studies exploring ultrasound-based radiomic models for risk stratification did not demonstrate clear superiority over conventional assessment methods [16]. Although current findings are not yet definitive, AI-based imaging approaches remain promising. Further well-designed, prospective, and externally validated studies are required to improve methodological quality, reproducibility, and clinical applicability. MRI-based radiomic analysis has demonstrated promising predictive performance in the preoperative assessment of EC, particularly for tumor grading, deep myometrial invasion, lymphovascular space invasion, and other high-risk pathological features [17-19]. A recent systematic review and meta-analysis evaluating MRI-based radiomics in EC included 45 studies and reported promising diagnostic and risk prediction performance. Radiomic models demonstrated good accuracy for differential diagnosis and preoperative risk assessment. However, the overall methodological quality of the included studies was moderate to low, with significant concerns regarding standardization, reproducibility, and external validation. These limitations currently restrict the routine clinical implementation of MRI radiomics in EC [20]. The definitive diagnosis of EC is established through histopathological examination of endometrial tissue obtained by Pipelle biopsy or D&C [2]. Recent advances in digital pathology, enabled by high-resolution whole-slide imaging (WSI), have improved diagnostic standardization, reproducibility, and opportunities for quantitative analysis. However, the interpretation of digitized slides may require longer review times compared with conventional light microscopy, particularly during the initial phases of implementation [21,22]. Novel multimodal approaches combining biomarker profiling and AI are being investigated to enhance non-invasive detection of EC. In a study by Njoku et al., protein biomarkers extracted from blood and cervicovaginal fluid samples were analyzed in 118 symptomatic postmenopausal women. Using machine learning techniques, the authors identified a subset of highly discriminative proteins associated with early-stage (International Federation of Gynecology and Obstetrics (FIGO) I) disease, supporting the feasibility of integrating molecular signatures into AI-driven detection models [23]. Molecular profiling has become a cornerstone of EC management. Genomic sequencing and gene expression analyses enable the identification of key molecular alterations involved in cell proliferation, DNA repair, and apoptosis, thereby refining risk stratification and guiding personalized therapeutic strategies [2]. Recent advances suggest that AI may further enhance this paradigm by predicting molecular subtypes directly from histopathological images. Multiple studies have shown that deep learning algorithms are capable of predicting underlying molecular alterations directly from hematoxylin and eosin (H&E)-stained histological slides [24]. In this context, Fremond et al. developed and validated the first deep learning model (im4MEC) designed to predict EC molecular classification using routine H&E slides. The study included 2,028 patients enrolled in the PORTEC-1, PORTEC-2, and PORTEC-3 clinical trials. By integrating morphological and molecular data, the authors demonstrated that their model could reliably predict molecular subtypes based solely on histopathological features, supporting the feasibility of AI-driven morpho-molecular classification in EC [25].

Applicability of AI in staging

Accurate identification of lymph node involvement plays a pivotal role in guiding adjuvant treatment strategies and determining overall prognosis. The risk of nodal metastasis ranges from approximately 5% to 40%, depending on key pathological factors such as histological subtype, tumor grade, and depth of myometrial invasion. In recent years, AI has emerged as a promising tool to refine risk stratification for lymph node metastasis. By integrating clinicopathological and imaging data, AI-driven models can enhance predictive accuracy, support individualized decision-making, and facilitate more tailored therapeutic approaches [26]. Preoperatively, the evaluation of myometrial invasion primarily relies on pelvic MRI. However, the accuracy of image interpretation depends on the expertise and experience of the radiologist, which can vary between individuals. A systematic review analyzed eight published studies evaluating different AI systems for predicting the depth of myometrial invasion based on MRI data. AI systems have demonstrated better sensitivity and specificity in assessing myometrial invasion on pelvic MRI images, leading to a more objective and standardized approach, ultimately improving EC management. However, results were influenced by other parameters, such as the coexistence of uterine fibroids, which could distort AI predictions, and differences in radiomic criteria used between studies [27]. Preoperatively, assessment of myometrial invasion is primarily based on pelvic MRI. However, the diagnostic performance of MRI remains dependent on the radiologist’s expertise and interpretative experience, resulting in potential interobserver variability. Petrila et al. evaluated in their systematic review eight studies investigating AI-based models designed to predict the depth of myometrial invasion using MRI data. Overall, AI systems demonstrated improved sensitivity and specificity compared with conventional image interpretation, supporting a more objective, reproducible, and standardized evaluation of myometrial infiltration. Such advances may enhance preoperative risk stratification and optimize therapeutic decision-making in EC. Nevertheless, model performance was influenced by several confounding factors, including the presence of concomitant uterine fibroids, which may alter uterine anatomy and compromise predictive accuracy. In addition, heterogeneity in radiomic feature extraction and methodological approaches across studies limits direct comparability and underscores the need for further validation and standardization [27]. Another recently published study proposed a deep learning pipeline based on MRI images to predict myometrial and cervical stromal invasion preoperatively. This study developed a deep learning pipeline based on sagittal T2-weighted MRI to automatically segment the uterus and tumor and predict deep myometrial and cervical stromal invasion in EC. A total of 178 patients were included, with model performance evaluated using a threefold stratified validation and compared to expert radiologic assessment. The model achieved balanced accuracies of 0.702 for deep myometrial invasion and 0.721 for cervical stromal invasion, compared with 0.769 and 0.859 for expert evaluation, respectively. Segmentation performance yielded Dice scores of 0.847 for the uterus and 0.579 for the tumor. Although challenges such as data variability, class imbalance, and imaging artifacts remain, this automated framework demonstrates strong potential as a supportive tool for preoperative staging. Its consistent performance in delineating the uterus and tumor regions further emphasizes its relevance for improving MRI-based evaluation in clinical practice [28]. More recently, a meta-analysis evaluated the diagnostic performance of AI-based MRI for the preoperative prediction of deep myometrial and cervical stromal invasion in EC. Eight studies (13 cohorts) were included after quality assessment using the Quality Assessment of Diagnostic Accuracy Studies-2 (QUADAS-2) tool. Pooled results demonstrated that AI-assisted MRI achieved a sensitivity of 0.80 and specificity of 0.81 (AUC 0.83) for predicting deep myometrial invasion. For cervical stromal invasion, diagnostic performance was even higher, with a sensitivity of 0.78, specificity of 0.86, and an area under the curve (AUC) of 0.90. Overall, these findings suggest that AI-enhanced MRI may improve the accuracy of preoperative staging in EC. Nevertheless, further large-scale, prospective, multicenter studies are required to confirm its clinical utility [29].

Applicability of AI in treatment

The management of EC is based on a multimodal approach tailored to tumor stage, histopathological characteristics, and molecular classification. Surgery remains the cornerstone of treatment, typically consisting of total hysterectomy with bilateral salpingo-oophorectomy, and lymph node assessment when indicated. Additional modalities, including radiotherapy, chemotherapy, hormonal therapy, or combined strategies, may be required depending on individual risk factors. In the postoperative setting, adjuvant therapy is determined according to international risk stratification systems incorporating FIGO stage, tumor grade, lympho-vascular space invasion, histologic subtype, and molecular profile. Options range from surveillance in low-risk patients to radiotherapy, chemotherapy, or combined treatments in higher-risk groups. Accurate prognostic evaluation is essential to guide adjuvant decisions and avoid overtreatment [2]. In this context, AI may enhance individualized risk prediction by integrating clinical, imaging, and molecular data, thereby supporting more precise therapeutic strategies. AI has primarily been applied in EC management to support intraoperative decision-making, particularly in the context of robotic-assisted surgery. In contrast, its role in guiding postoperative adjuvant treatment selection remains insufficiently explored, with only a limited number of studies addressing this issue. Mysona et al. developed a predictive nomogram to estimate the benefit of adjuvant chemotherapy in patients with stage IA serous papillary EC. Using demographic and surgical data from 1,751 patients, the authors constructed a prognostic model based on a random survival forest machine learning algorithm. This approach led to the development of a web-based application intended to facilitate risk stratification and potentially inform future clinical research and therapeutic decision-making [30]. Another study classified EC into subgroups using a machine learning model to tailor treatment protocols, thereby assessing response to chemotherapy and immunotherapy based on AI models [31]. The same research team that developed the deep learning model im4MEC for molecular classification prediction of EC from H&E WSIs [32] also developed a multimodal deep learning prognostic model called HECTOR (Histopathology-based Endometrial Cancer Custom Outcome Risk). HECTOR is a deep learning model trained and validated in stage I-III EC, predicting distant recurrence risk in surgically treated patients using only H&E-stained tumor slides and anatomical staging. Tumor data from 2,072 patients across eight cohorts, including those from randomized trials PORTEC-1, PORTEC-2, and PORTEC-3, were used. The primary objective of this model was to predict distant recurrence in EC. Molecular classification of EC currently holds major prognostic significance. p53-abnormal (p53abn) ECs are considered high-risk tumors, while mismatch repair-deficient (MMRd) and no specific molecular profile (NSMP) ECs are classified as intermediate-risk tumors. HECTOR could refine prognosis within molecular subtypes, particularly among low-risk groups. HECTOR identified 16 patients (5.3%) within the p53abn group who had a favorable prognosis. This model also impacts therapeutic decision-making, such as the addition of adjuvant chemotherapy to external beam radiotherapy. Validation of the HECTOR model in a prospective clinical trial could improve therapeutic decision-making because predicting recurrence risk based on HECTOR-Risk groups would allow for treatment de-escalation or escalation and ensure a faster and cost-effective precision treatment strategy [32].

Applicability of AI in prognosis

A wide range of AI-based models have been developed for prognostic prediction across multiple oncologic domains, including lung, breast, and colorectal cancers. In contrast, the application of AI-driven prognostic tools in EC remains comparatively limited, with relatively few studies addressing this area to date. AI-based applications in medical imaging have also emerged as valuable tools for prognostic assessment in EC. Qi proposed an AI model to evaluate the role of deep learning combined with MRI in prognostic prediction in EC. An optimized convolutional NN (ResNet-101) incorporating spatial and channel attention mechanisms was trained on MRI data from 210 patients. The proposed model demonstrated significantly superior performance compared with conventional architectures in identifying high-risk patients, achieving an AUC of 0.918. Importantly, it also showed excellent accuracy in predicting postoperative recurrence (AUC 0.926), clearly outperforming standard models. These findings highlight the potential of AI-driven MRI analysis as a powerful tool for prognostic stratification and recurrence risk prediction in EC [33]. More recently, a machine learning-based study explored the use of proteomic data to stratify prognostic factors in EC. Using tumor biopsy samples from 20 patients, the authors applied different machine learning algorithms (XGBoost and LightGBM) combined with explainable AI (SHAP) to classify key prognostic variables, including age, tumor grade, myometrial invasion, and tumor size. The models demonstrated high discriminatory performance, with AUC values ranging from 94.8% to 98.8% depending on the variable analyzed. Beyond classification accuracy, the integration of explainable AI enabled the identification of potential protein biomarkers associated with these prognostic factors, with EWRS1 emerging as a recurrent marker across multiple subgroups. These findings suggest that AI-driven proteomic analysis may contribute to more refined prognostic stratification in EC while also highlighting candidate biomarkers for future validation [34]. Other machine learning algorithms have been developed using clinical, demographic, and histological data to predict cancer-specific survival (Table 1) [35,36].

**Table 1:** Landmark studies investigating AI applications in EC management AI: artificial intelligence; EC: endometrial cancer; LR: logistic regression; RF: random forest; SVM: support vector machine; NN: neural network; DT: decision tree; LDA: linear discriminant analysis; NB: naïve Bayes; EIN: endometrial intraepithelial neoplasia; BMI: body mass index; ANN: artificial neural network; CART: classification and regression tree; CNN: convolutional neural network; DL: deep learning; ML: machine learning; MRI: magnetic resonance imaging; LVSI: lymphovascular space invasion; CAD: computer-aided diagnosis; WSI: whole-slide imaging; H&E: hematoxylin and eosin; Se: sensitivity; Sp: specificity; FIGO: International Federation of Gynecology and Obstetrics

Reference	Clinical domain	Study design	Study population	AI methodology	Input data	Primary endpoint	Performance
Troisi et al., 2020 [4]	Screening	Validation study	1,550 postmenopausal women	ML	Serum metabolomic signature	Detection of EC	Se > 99%; Sp > 99%
Hart et al., 2020 [5]	Risk prediction	Population-based	Large community cohort	LR, RF, SVM, NN, DT, LDA, NB	Clinical variables	5-year EC risk prediction	RF and NN highest performance
Kitson et al., 2017 [6]	Prevention	Predictive model	High-risk women	ML	Clinical risk factors	Risk stratification	Promising
Erdemoglu et al., 2023 [11]	Diagnosis	Retrospective	564 women	Boruta + ML	Age, BMI, endometrial thickness	Prediction of EIN/EC	High accuracy
Pergialiotis et al., 2018 [12]	Diagnosis	Diagnostic study	Postmenopausal women	ANN & CART	Clinical + ultrasound	EC prediction	Good performance
Zhang et al., 2021 [14]	Hysteroscopy	Image classification	454 patients (1,851 images)	VGGNet-16 CNN	Hysteroscopic images	Lesion classification	Outperformed gynecologists
Lecointre et al., 2021 [15]	Radiomics	Systematic review	17 studies	ML/DL	MRI & ultrasound	Diagnostic performance	Promising
Moro et al., 2022 [16]	Ultrasound radiomics	Validation	EC patients	Radiomics	Ultrasound	High-risk prediction	Moderate
Di Donato et al., 2023 [17]	MRI radiomics	Meta-analysis	Multiple cohorts	Radiomics	MRI	Grade/invasion/nodes	Good pooled accuracy
Huang et al., 2024 [20]	MRI radiomics	Meta-analysis	Multiple studies	Radiomics	MRI	Diagnostic performance	Confirmed utility
Meng et al., 2024 [19]	MRI	Meta-analysis	Multiple studies	AI-assisted MRI	MRI	LVSI prediction	Moderate
Fell et al., 2023 [21]	Digital pathology	Development	WSI	DL	Histopathology	Malignancy detection	Improved diagnosis
Vermorgen et al., 2024 [22]	Digital pathology	Feasibility	Pipelle biopsies	CAD	Histopathology	Benign vs malignant	Feasible
Njoku et al., 2024 [23]	Biomarkers	Prospective	118 women	ML	Blood + cervicovaginal proteomics	Early EC detection	Discriminative signature
Darbandsari et al., 2024 [24]	Molecular pathology	Translational	Digital pathology cohort	AI histopathology	WSI	Subtype prediction	High accuracy
Fremond et al., 2023 (im4MEC) [25]	Molecular classification	Multicenter	2,028 patients	DL	H&E WSI	Molecular classification	High concordance
Petrila et al., 2023 [27]	Staging	Systematic review	8 studies	ML	MRI	Myometrial invasion	Higher Se/Sp
Lecointre et al., 2025 [28]	Staging	Development	MRI cohort	DL segmentation	MRI	Tumor segmentation	Excellent
Mysona et al., 2020 [30]	Treatment	Retrospective	1,751 patients	Random survival forest	Clinical + surgical	Chemo benefit	Validated nomogram
Lu et al., 2023 [31]	Treatment	Molecular	EC cohort	ML	Clinical + molecular	Therapeutic subgroups	Promising
Volinsky-Fremond et al., 2024 [32]	Prognosis	Multicenter	2,072 patients	Multimodal DL	H&E + FIGO	Recurrence prediction	Superior to conventional
Qi et al., 2024 [33]	Prognosis	Imaging	MRI cohort	DL	MRI	Recurrence prediction	Improved
Yasar et al., 2024 [34]	Prognostic biomarkers	Proteomic	EC patients	Explainable AI + ML	Proteomics	Biomarker discovery	Robust
Shazly et al., 2023 [35]	Prognosis	Validation	EC patients	ML	Clinicopathological	Survival prediction	Good discrimination
Praiss et al., 2020 [36]	Prognosis	Multicenter	EC patients	ML	Clinical + pathological	Cancer-specific survival	Superior

Challenges and limitations of AI applications

The development of robust AI models requires access to large, high-quality, and well-annotated datasets. Such resources remain limited in many low- and middle-income countries, where electronic medical record systems are not fully implemented. Insufficient data quality and representativeness may compromise model performance and generalizability, increasing the risk of algorithmic bias and inequitable predictions. Despite growing interest in AI, resistance to its clinical adoption persists among some healthcare professionals. This underscores the importance of structured training programs, transparent model design, and rigorous external validation to enhance trust and facilitate integration into routine practice. Importantly, AI should be viewed as a complementary decision-support tool rather than a replacement for clinical expertise. Human-machine collaboration has the potential to optimize diagnostic accuracy, prognostic assessment, and therapeutic decision-making in EC, ultimately improving patient outcomes and quality of life. Ethical considerations also represent a major challenge. The implementation of AI systems requires strict governance frameworks to ensure data privacy, confidentiality, and compliance with regulatory standards. Furthermore, compared with cervical cancer, AI applications in EC remain relatively underdeveloped. This is largely due to limited data availability, smaller cohort sizes, and the lack of external validation for many published models. The absence of standardized methodologies and reporting frameworks further complicates cross-study comparisons and hinders the translation of AI research into clinical practice.

## Conclusions

In comparison with other gynecologic malignancies, the literature exploring AI applications in EC remains limited. The currently available evidence is insufficient to support the development of standardized clinical recommendations or formal integration into guidelines. Nonetheless, AI represents a highly promising component of precision medicine, with potential applications spanning the entire continuum of EC management, from early detection and risk assessment to therapeutic planning and outcome prediction. In low-income settings, where access to subspecialists and comprehensive histopathological services is often restricted, AI may serve as a practical and economically sustainable diagnostic support tool. By enhancing image interpretation and assisting clinical decision-making, AI-based systems could help reduce diagnostic delays, optimize the allocation of limited healthcare resources, and contribute to narrowing disparities in cancer care delivery.
